# Strawberry Achenes Are an Important Source of Bioactive Compounds for Human Health

**DOI:** 10.3390/ijms17071103

**Published:** 2016-07-11

**Authors:** María Teresa Ariza, Patricia Reboredo-Rodríguez, Luca Mazzoni, Tamara Yuliett Forbes-Hernández, Francesca Giampieri, Sadia Afrin, Massimiliano Gasparrini, Carmen Soria, Elsa Martínez-Ferri, Maurizio Battino, Bruno Mezzetti

**Affiliations:** 1 Instituto Andaluz de Investigación y Formación Agraria y Pesquera (IFAPA), Consejería de Agricultura, Pesca y Desarrollo Rural, Junta de Andalucía, IFAPA de Churriana, Cortijo de la Cruz s/n, Churriana, 29140 Málaga, Spain; maria.soria@juntadeandalucia.es (C.S.); elsa.martinez@juntadeandalucia.es (E.M.-F.); 2 Área de Nutrición y Bromatología, Departamento de Química Analítica y Alimentaria, Universidad de Vigo, Campus de Ourense, E-32004 Ourense, Spain; preboredo@uvigo.es; 3 Dipartimento di Scienze Agrarie, Alimentari e Ambientali, Università Politecnica delle Marche, Ancona 60131, Italy; l.mazzoni@live.it; 4 Department of Clinical Sciences, Faculty of Medicine, Polytechnic University of Marche, Ancona 60131, Italy; tamara.forbe@gmail.com (T.Y.F.-H.); f.giampieri@univpm.it (F.G.); dolla.bihs@gmail.com (S.A.); m.gasparrini@univpm.it (M.G.); m.a.battino@univpm.it (M.B.)

**Keywords:** in vitro digestion, fruit, seeds, breeding, bioaccessibility, phenolics, flavonoids, anthocyanins, antioxidant capacity

## Abstract

Strawberries are highly appreciated for their taste, nutritional value and antioxidant compounds, mainly phenolics. Fruit antioxidants derive from achenes and flesh, but achene contribution to the total fruit antioxidant capacity and to the bioaccessibility after intake is still unknown. In this work, the content of total phenolic compounds, flavonoids, anthocyanins and antioxidant capacity (TEAC, FRAP and DPPH) of achenes and flesh were compared in non-digested as well as in gastric and intestinal extracts after in vitro digestion. Results showed that, despite strawberry achenes represent a small fraction of the fruit, their contribution to total fruit antioxidant content was more than 41% and accounted for 81% of antioxidant capacity (TEAC). Achenes have higher quantity and different quality of antioxidants in non-digested and digested extracts. Antioxidant release was higher in the in vitro gastric digested extracts, but digestion conditions did not only affect quantity but quality, resulting in differences in antioxidant capacity and highlighting the importance of simulating physiological-like extraction conditions for assessing fruit antioxidant properties on human health. These results give new insights into the use of strawberry achenes as a source of bioactive compounds to be considered in strawberry breeding programs for improving human health.

## 1. Introduction

Strawberries (*Fragaria × ananassa*, Duch.) are among the most widely consumed fruits in the world. They are a very rich source of bioactive compounds including vitamins (such as vitamin C), β-carotene and phenolic compounds (phenolic acids, flavonoids and anthocyanins) [1]. These bioactive compounds, such as antioxidants, reduce oxidative stress by neutralizing the overproduction of reactive oxygen species (ROS), which are related with the occurrence of several diseases [2,3].

In epidemiological studies, strawberry consumption has been associated with health benefits [4,5,6], such as the prevention of inflammation [7], oxidative stress [8,9], cardiovascular diseases [10,11], diabetes [12], cancer [13,14] and obesity [15]. These healthy effects have been related with the antioxidant activity of phenolic compounds, mainly ellagitannins [13,14] and anthocyanins [16,17,18,19,20,21,22,23,24,25,26]. 

Healthy properties of strawberries are not only associated with the amount of bioactive compounds, but also with the degree of transformation during digestion. In fact, in strawberry fruits, the chemical nature and/or the integrity of bioactive compounds can be altered by the specific conditions of the gastrointestinal tract, the activity of gastrointestinal enzymes [27] and by the action of the local microbiota [28], affecting their uptake throughout the digestive tract [29]. For example, a major part of the polyphenols ingested is not absorbed through the gut barrier [30], while anthocyanins are directly and quickly absorbed from the stomach [31,32,33] and from the small intestine [28,34,35].

The so-called strawberry is actually an aggregate fruit consisting of a swollen and fleshy floral receptacle that supports a cluster of real dry fruits or achenes containing the seeds [36]. It has been reported that phenolic composition in achenes and flesh differs [17,37], achenes having up to 10-fold higher antioxidant activity and amount of phenolic compounds, especially ellagic acid derivatives [17,38]. In this sense, achenes could themselves represent a significant source of bioactive compounds for human health. However, after intake, achene contribution to the antioxidant capacity of the whole fruit is unknown and could be negligible due to the barrier interposed by their hard and relatively thick pericarp with ligno-cellulosic structure [39].

In the present study, we have compared strawberry phenolic composition and antioxidant capacity between achenes and raw fruit, before and after simulated in vitro digestion, in order to characterize and quantify the release and bioaccessibility of phenolic compounds in both types of tissues.

## 2. Results

### 2.1. Achene Contribution to Total Fruit Weight

Fresh weight of “Camarosa” strawberry fruits was 21.9 ± 0.8 g and achenes represented 0.75% of total fresh weight (i.e., 0.16 ± 0.02 g). Considering that 90% of total fresh fruit weight is water [17], achenes would then represent the 7.5% of total fruit dry weight. These figures were used to calculate the relative contribution of achenes and flesh to antioxidant content and capacity in a whole fruit (see Table 1).

### 2.2. Antioxidant Composition in Non-Digested Raw Fruits and Achenes

**Figure 1:**
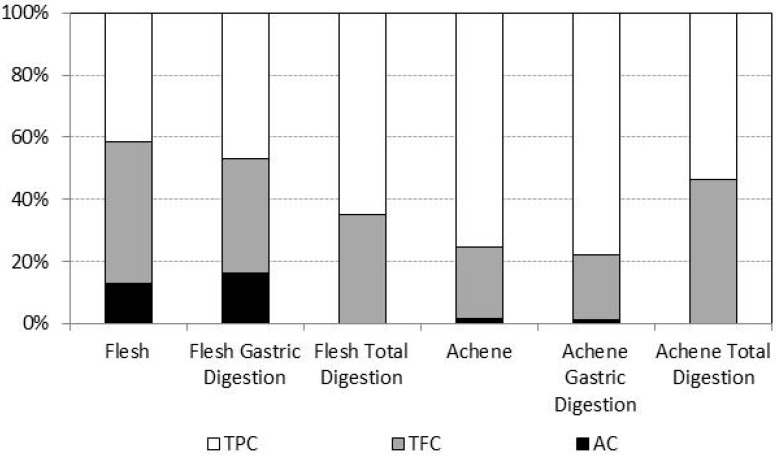
Relative contribution of total flavonoids (TFCs) and anthocyanins (ACs) to total content of phenolic compounds (TPCs) in six different extracts (non-digested flesh, non-digested achenes, flesh and achenes after gastric digestion and flesh and achenes after total digestion).

There were differences in the levels of antioxidant compounds between non-digested flesh and achenes. Achenes displayed significantly higher total phenolic compounds (TPCs) and total flavonoid compounds (TFCs) than flesh (more than 10 and four times higher, respectively; see Table 2). No significant differences were found between flesh and achenes in anthocyanin content (AC). The differences in antioxidant composition in achenes and flesh translated into a different contribution of flavonoids and anthocyanins respect to TPC (Figure 1). Thus, TFCs were 44.15% and AC 14.33% of TPC in flesh, whereas in achenes, these percentages were 22.71% and 1.82%, respectively. In addition, antioxidant capacity analyzed by FRAP, DPPH and TEAC was, respectively, 17-, 12- and 64-times higher in achenes than in flesh (Table 2).

Although achenes showed a higher amount of TPCs and TFCs (Table 2), there were differences in the composition of individual compounds between flesh and achenes. Phenolic acids (caffeic, chlorogenic and ellagic acids) and anthocyanins (Cya 3-glc, Pel 3-glc and Pel 3-rut) analyzed by HPLC represented 22% and 4% of TPC in flesh and achenes, respectively (Table 3). The amount of caffeic and chlorogenic acid was significantly higher in flesh than in achenes, whereas the reverse was true for ellagic acid. However, although in the case of Pel 3-rut no significant differences were found, Cya 3-glc and Pel 3-glc displayed higher values in achenes (three-fold and 2.1-fold, respectively; see Table 3).

It is remarkable that, despite the achenes representing only 7.5% of the total fruit dry weight, their relative contribution to the total antioxidant content and capacity of the whole fruit is quite high (Table 1), accounting for 41% of TPC and 23% of TFC and even up to 81% of the fruit antioxidant capacity.

### 2.3. Bioaccessibility of Antioxidant Compounds after in Vitro Digestion

Flesh and achenes differed in TPC, AC, FRAP, DPPH values in the gastric fraction (Table 2). Hence, TPC, FRAP and DPPH data were more than two-, three- and 32-fold, respectively, higher in achenes compared with flesh, but the reverse was true for AC (two-fold higher in flesh). However, in the intestinal fraction, the opposite tendency was observed (Table 2). Antioxidant compounds and antioxidant capacity in both flesh and achenes were higher in gastric than in intestinal fractions (Table 2). Thus, the recovery after total digestion of TPC, TFC and AC was 16.1%, 10.6% and 0%, in flesh and 2.8%, 5.7% and 0% in achenes, respectively.

In addition, in gastric fraction, antioxidant capacity by FRAP and DPPH assays was higher in achenes than in flesh (Table 2), whereas, for TEAC, no differences were found. In intestinal fraction, flesh showed significantly higher values of antioxidant capacity than achenes (Table 2). When gastric and intestinal fractions were compared, the antioxidant capacity in the intestinal fractions from both flesh and achenes was decreased (Table 2). Thereby, FRAP, DPPH and TEAC was reduced by 92%, 24% and 87% in flesh and by 98%, 99% and 96% in achenes, respectively.

When flesh and achenes were subjected to gastric digestion, no significant differences were observed in the caffeic, chlorogenic and ellagic acid content (Table 3). However, the amount of anthocyanins (Cya 3-glc, Pel 3-glc and Pel 3-rut) was significantly higher in achenes than in flesh. In both flesh and achenes, there was a major trend to lower values of phenolic acids and anthocyanins in the intestinal fraction except for caffeic acid in achenes (Table 3). The decrease of phenolic acids was more marked in flesh than in achenes, resulting in lower percentages of recovery. It is remarkable that Cya 3-glc in flesh and Pel 3-rut in achenes were not detected after intestinal digestion.

### 2.4. Relationship between Antioxidant Compounds and Antioxidant Capacity

**Figure 2:**
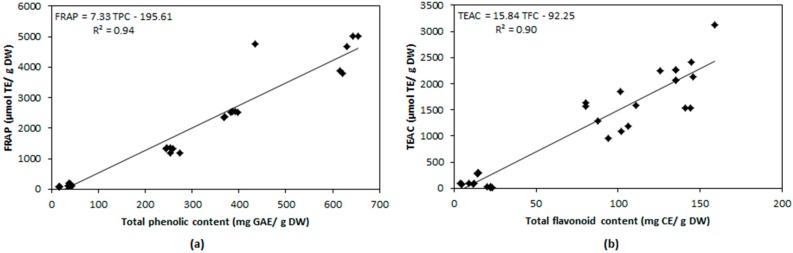
Linear relationship between total phenolic content and FRAP (**a**) and total flavonoid content and TEAC (**b**).

To assess the relationship between variables, data from non-digested and digested extracts (i.e., gastric and intestinal fraction) from flesh and achenes were analyzed jointly. Most of the parameters evaluated were highly and significantly correlated (Table 4). However, no correlation was found between AC and FRAP and DPPH. Regression analysis showed that most of the variation observed in FRAP was absorbed by TPCs (*R*^2^ = 94.4%; Figure 2a), of which TFCs accounted for 64% and ACs did not contribute. Variation in TEAC was mainly explained by TFCs (*R*^2^ = 90%; Figure 2b), and ACs accounted for 54%. Variation in DPPH was partially related with TPCs and TFCs (*R*^2^ = 51% and 15%, respectively).

### 2.5. Efficiency of Extraction Conditions

Taking into consideration that extraction conditions could affect antioxidant release, the comparison between non-digested and gastric extracts is interesting.

The majority of compounds analyzed in flesh and achenes were released to a greater extent after gastric digestion in comparison with non-digested extracts. This translated into values of extraction efficiency (Ee) far above one (Table 2), especially in flesh.

In relation to specific compounds, the release of anthocyanins (Cya 3-glc, Pel 3-glc and Pel 3-rut; Table 3) was higher in the gastric extract, whereas the reverse was true for specific phenolic acids (caffeic, chlorogenic and ellagic acids), in both flesh and achenes.

## 3. Discussion

The present study gives new insights into the relevance of strawberry achenes as a source of antioxidant and bioactive compounds providing a new profitable product for human health.

As reported in previous studies [17,37], our results showed that strawberry antioxidant properties are linked either to flesh or to achenes. Despite achenes representing a small fraction of total fruit weight, their contribution to total antioxidant content and capacity of the fruit is remarkable. Although in the whole fruit, phenolic compounds, flavonoids and anthocyanins are mainly in the flesh, we found that strawberry antioxidant capacity (i.e., FRAP and TEAC assays) was mainly attributable to achenes, suggesting that not only the amount of antioxidants, but mostly the type, accounts for strawberry ROS detoxification capacity. In fact, although achenes were enriched in phenolic compounds (including flavonoids) compared to flesh, in consonance with a higher antioxidant capacity (FRAP; DPPH and TEAC), only 20% of total phenolic compounds were flavonoids, whereas, in the flesh, flavonoids represented 60%, indicating that non-flavonoid phenolic compounds are preferably in the achenes. Similarly, anthocyanins were mostly in the flesh. Even more, specific phenolics and anthocyanins are not equally present in flesh and achenes, suggesting some degree of compartmentation of antioxidant compounds in the strawberry fruit. These results are in agreement with the higher antioxidant content of seeds in comparison to the edible part of fruits [40,41].

The differences between flesh and achenes in antioxidant composition and capacity might be due to several factors, such as the nature of the matrix where the antioxidants are embedded. Intrinsically achenes have more antioxidant compounds than flesh, but different matrices can determine the effectiveness of digestive enzymes, and, therefore, affect the release of bioactive compounds under the physiological conditions during the digestion [42]. In this sense, the amount of antioxidants in gastric digested flesh, and achenes were quite higher than in non-digested samples, suggesting that in vitro digestion provide better conditions (i.e., low pH and pepsin; [43]) for the release of phenolic compounds from the matrix and/or for their stability [44]. These results are pointing out that phenolic quantification in non-digested extracts of strawberries is underestimated by the extraction methods commonly used. Nevertheless, gastric conditions favored a greater release of antioxidants in the flesh than in achenes (i.e., higher Ee), indicating that the matrix exerts a differential resistance to the release of antioxidant compounds. However, gastric conditions do not always translate into a higher release of specific compounds, such as caffeic, chlorogenic and ellagic acids, pointing to chemical transformation and/or degradation. In contrast, the specific anthocyanins analyzed were increased in gastric fraction probably due to higher stability under low pH [45,46].

A drastic decrease of phenolic compounds and flavonoids was observed in the intestinal fraction, but, in agreement with Clifford [47], anthocyanins were completely absent. The low amount of phenolic compounds indicates that the intestine imposes a limitation to their availability that can result either from chemical modifications or physical barriers [48]. Chemical effects could arise from antioxidant degradation or from alteration of the antioxidant properties caused by intestinal conditions (i.e., in our study, pH 7.7, bile salts, and pancreatin) [49] since the action of the local microbiota is not considered in this study, although it contributes significantly to the digestive process.

Physical barriers, represented by the dialysis membrane, can limit the uptake of high molecular weight compounds [50]. Although chemical and physical aspects might be interacting at the intestine level determining the recovery in the bloodstream, our results in specific compounds (phenolic acids and anthocyanins) are pointing out that this effect is complex and depends on each molecule. Thus, not all the phenolics showed the same percentage of recovery, reaching up to 236% for caffeic acid in achenes, indicating that molecule reassembly could be occurring. This result might be relevant for human health, since it suggests that beneficial compounds could derive from the distinct precursors provided by the food matrices.

Nevertheless, the low rate of antioxidant recovery in the intestinal fraction does not necessarily involve a low absorption, since some phenolic compounds, such as anthocyanins, can be absorbed through the gastric wall [28,32,33]. Even more, in vivo models have revealed that antioxidants can pass through the gut membrane either by passive diffusion or by specific membrane transporters after the interaction with certain glucosides (active glucose transporter: SGLT1) [47]. Therefore, antioxidant absorption could be underestimated after in vitro digestion.

In both achenes and flesh, the amount of antioxidants was closely related with the antioxidant capacity: the higher the antioxidants, the higher the antioxidant capacity at all levels during the digestive process. However, not all antioxidant compounds contribute equally to the total antioxidant capacity and are not similarly involved in the different radical scavenging mechanisms. Thus, the FRAP method detects exclusively electron transfer mechanisms (SET), whereas TEAC and DPPH combine SET and Hydrogen Atom Transfer (HAT) mechanisms [51]. In contrast to other reports attributing antioxidant capacity mainly to anthocyanins [52], our results showed that anthocyanins only contribute 54% to TEAC but neither to DPPH (in disagreement with Espin et al. [53]) nor to FRAP, suggesting that anthocyanins antioxidant activity should be associated to HAT mechanisms. On the other hand, the high correlation between FRAP and total phenolic compounds (that include flavonoids) indicate that SET mechanisms are mainly related either to non-flavonoids (~30%) and non-anthocyanins flavonoid (~60%) phenolic compounds. Moreover, the minimal relationship between DPPH and total phenolic compounds (50%) and total flavonoids (15%) is indicative of its dependence on both non-flavonoids phenolic compounds (~35%) and other non-phenolic antioxidants (~50%).

In summary, this work highlights not only the relevance of achenes in the strawberry antioxidant capacity, but their potential healthy properties under physiological-like conditions. These results give new insights for strawberry profitability and open a wide range of applications for improving human health (i.e., pharmaceutical uses). Thus, achenes might become a valuable product representing a new concept to be introduced in strawberry cultivation and breeding programs. In this sense, some strawberry varieties, which, at present, have been discarded because of misshapen fruits (i.e., “Camarosa”), could become interesting for achene production [54]. Moreover, achene antioxidant composition can be introduced as a new feature in breeding programs for characterization of strawberry cultivars, as for raw fruits [55], and would confer a differential healthy value to each variety. Apart from the healthy benefits, the use of achenes as a commercial/profitable product would allow the utilization of industrial processing waste [17] and would also entail great advantages to producers, since it is a non-perishable fruit, with easy conservation and transportation, and suitable to be added to different types of food.

## 4. Materials and Methods

### 4.1. Strawberry Material and Sample Preparation

Strawberries (*Fragaria × ananassa* Duch cv. Camarosa) were planted in mid-October 2014 at the IFAPA-Churriana experimental greenhouse (Málaga, Spain). Three replications of 50 fresh and fully-ripened strawberry fruits were harvested. Fruit weight was calculated and all the achenes per fruit were manually separated from the flesh and weighed. Afterwards, both fractions were lyophilized by Labconco Freezone 12 L mod. 78670 (Kansas City, MO, USA). Flesh was ground into a fine powder with Mixer B-400 (Büchi Labortechnik, Flawil, Switzerland), and samples were stored at −20 °C until analysis.

To obtain the hydroalcoholic extracts for the analysis of phytochemicals content and total antioxidant capacity, 1 g of lyophilized sample (flesh or achenes) were added to 100 mL of 80% methanol aqueous solution acidified with 0.1% formic acid and stirred for 2 h in the dark at room temperature. Extracts were centrifuged at 3000 rpm for 15 min, and the supernatant was filtered and stored at −80 °C until analysis.

**Figure 3:**
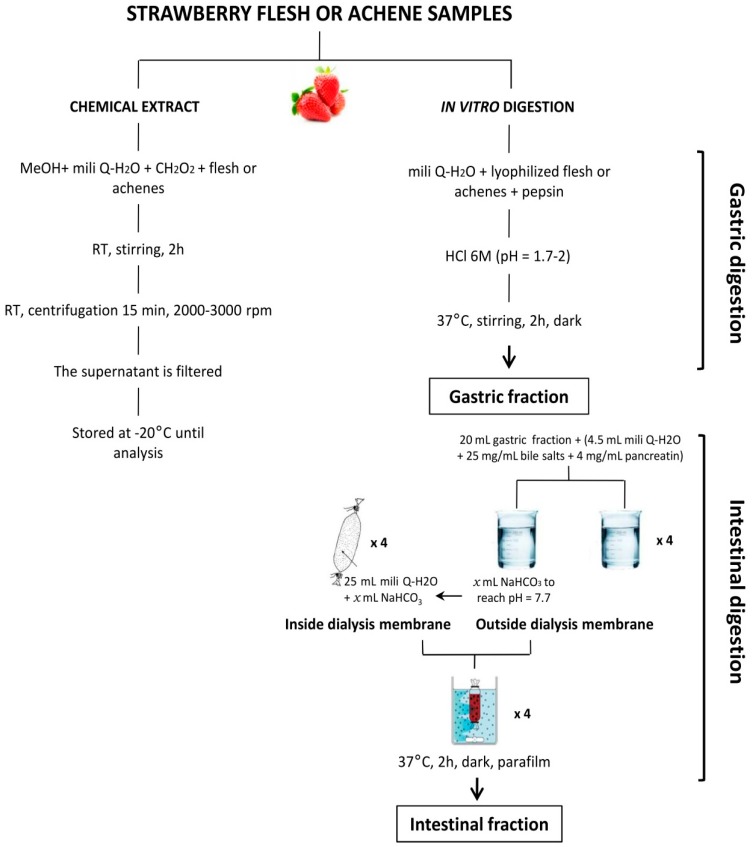
Scheme of the in vitro digestion general procedure used with strawberry and achene samples.

The in vitro digestion of lyophilized flesh and achenes was performed according to the method developed by Gil-Izquierdo et al. [42]. Briefly, the method consisted of an initial pepsin-HCl digestion for 2 h for simulating gastric conditions, (pH~1.8) followed by an intestinal digestion with pancreatin and bile salts for 2 h, both in a 37 °C shaking water bath. During the intestinal phase, a dialysis membrane (molecular weight cut-off of 12,000 Da, containing the amount of NaHCO_3_ necessary to titrate the mixture of post-gastric digestion fraction + pancreatin-bile to pH 7.8) was introduced in a beaker containing an aliquot of 20 mL of the post-gastric fraction (Figure 3). Gastric and intestinal fractions, from both raw fruit and achenes, were collected, concentrated under vacuum, purified (C-18 SepPaks Vac 6cc cartridge; Waters Assoc., Milford, MA, USA) and stored at −80 °C. Before analysis, gastric and intestinal fractions were opportunely re-suspended in a methanol: water solution and results were referred to the dried weight before re-suspension.

### 4.2. Measurement of Antioxidant Compounds

#### 4.2.1. Total Phenolic, Flavonoid and Anthocyanin Content

Total phenolic content was determined by the Folin–Ciocalteu method [56,57]. Briefly, 100 µL of samples or gallic acid standard solution were mixed with 500 µL of Folin–Ciocalteu reagent (10%), incubated for 3 min, and then 400 µL of sodium acetate solution (7 M) was added. After 2 h of incubation at room temperature in the dark, the absorbance at 760 nm was measured in a UV-Vis spectrophotometer (model DU^®^ 6400 Spectrophotometer, Beckman, Fullerton, CA, USA). Results were expressed as gallic acid equivalents per gram of dry weight (mg GAE/g DW).

Total flavonoid content was measured by a colorimetric method described by Dewanto et al. [58]. Briefly, 250 µL of samples or catechin standard solution were mixed to 1.25 mL of MilliQ water (Millipore, Bedford, MA, USA) and 75 µL of NaNO_2_ 5% solution. After 6 min, 150 µL of AlCl_3_·6H_2_O, 10% solution, was added, allowed to stand for 5 min, and then 500 µL NaOH 1 M were added. The volume was brought to 2.5 mL with water, and the absorbance was measured at 510 nm. Results were expressed as catechin equivalents per gram of dry weight (mg CAE/g DW).

Total anthocyanin content was measured by the pH differential method [59]. Briefly, 0.025 M potassium chloride (KCl) buffer, pH 1.0 (buffer 1) and 0.4 M sodium acetate buffer (CH_3_CO_2_Na), pH 4.5 (buffer 2) were prepared. After that, two dilutions (1/10 *v*/*v*) of the samples or pelargonidin-3-glucoside (Pel-3-glu) standard solutions were prepared, one with buffer 1 and the other with buffer 2. These dilutions were let to rest for 15 min, before measuring the absorbance of each extract at 500 and at 700 nm to correct for haze, against a blank represented by MilliQ water. The final absorbance (A) of the diluted samples was calculated as follows:

(1) $$A\;=\;\left[\left(A_{510}-\;A_{700}\right){\text{ pH}}_{1.0}-\;\left(A_{510}-\;A_{700}\right){\text{ pH}}_{4.5}\right]$$

Total anthocyanin content (AC) was calculated using the formulation reported by Cerezo et al. [60]:

(2) Total Anthocyanins = (A × MW × df × 1000)/(ε× 1)

where MW: Pel-3-glc molecular weight; df: dilution factor; ε: molar extinction coefficient of Pel-3-glc. Results were expressed as Pelargonidin-3-glucoside equivalents per gram of dry weight (mg Pel-glc/g DW).

#### 4.2.2. Total Antioxidant Capacity

Several methods for determination of antioxidant capacity were used: FRAP, DPPH, and TEAC assays. 

The FRAP (ferric reducing/antioxidant power) assay was carried out according to the protocol proposed by Deighton et al. [61]. The FRAP reagent solution was prepared as follows: 10:1:1 of sodium acetate (300 mM, pH 3.6), TPTZ (10 mM in HCl 40 mM) and ferric chloride (20 mM). Briefly, 100 μL of sample or Trolox standard solution were added to 900 μL FRAP reagent. The mixture was vortexed, and, after 4 min, the absorbance was read at 593 nm against blank. 

DPPH radicals (radical-scavenging activity) were determined based on the method described by Kumaran and Karunakaran [62]. Briefly, 50 µL of Trolox standard solution or samples were added to 1.450 mL of 3 mM DPPH solution. Subsequently, the mixture was vortexed and after 1 h of incubation into the dark, and its absorbance was read at 515 nm. 

TEAC (Trolox Equivalent Antioxidant Capacity) assay was performed according to Re et al. [63]. The ABTS radical solution was produced by reacting 7 mM ABTS aqueous stock solution with 2.45 mM K_2_S_2_O_8_, and maintained in the dark at 25 °C for 12 h before use. Immediately before analysis, the working solution was obtained by diluting the stock solution 1:50 with PBS buffer, pH 7.4. Briefly, 10 µL of alternatively blank, Trolox standard or MilliQ water diluted strawberry extract were added to 1 mL of the ABTS working solution into 1.5 mL eppendorfs. The mixture was vortexed for 20 s and after 1–3 min, the absorbance (A) was read at 734 nm, measuring the colour inhibition of the ABTS radical. The percentages of inhibition were calculated according the following equation:

(3) % inhibition = (A control − A sample/A control) × 100

FRAP, DPPH and TEAC results were expressed as Trolox equivalents per gram of dry weight (µmol TE/g DW).

### 4.3. Determination of Phenolic Acids and Anthocyanins by HPLC

Phenolics and anthocyanins were extracted by incubation of 7.5 mg of dry frozen powder in 300 µL of 96% methanol aqueous solution acidified with 0.001% formic acid, followed by centrifugation at 3000 rpm for 15 min, filtering and immediate analysis on a JASCO HPLC system (Jasco Corp., Tokyo, Japan) equipped with a quaternary gradient pump (JASCO PU-2089 Plus) and a multi-wavelength UV detector (JASCO UV-2070 Plus). System control and peak integration was done by the ChromNAV software (Jasco, Tokyo, Japan).

Samples were diluted appropriately and filtered using a 0.45 µm GHP Acrodisc Minispike filter (Pall Life Sciences, Ann. Arbor, MI, USA) and separated on an Aqua Luna C18 reverse phase column (250 mm × 4.6 mm; Phenomenex, Torrance, CA, USA) with a particle size of 5 μm protected by a C18 ODS guard column (4.0 mm × 3.0 mm; Phenomenex). The injection volume was set to 20 µL and the flow rate was 1.0 mL/min^−1^.

Phenolic acids were analysed following the method of Schieber et al. [64] with minor modifications. The mobile phase consisted of 2% (*v*/*v*) acetic acid in Milli-Q water (eluent A) and of acetic acid in water and acetonitrile (1:49:50, *v*/*v*/*v*; eluent B). The gradient program was as follows: 10% B to 55% B (50 min), 55% B to 100% B (10 min), 100% B to 10% B (1 min), and 10% B for 5 min before injecting the next sample. The phenolic acids were quantified using calibration curves from standards of chlorogenic, caffeic and ellagic acids. Values were expressed as milligrams of the corresponding phenolic acid per gram of dry weight (mg/g DW).

Analysis of anthocyanins was carried out following the method described in Fredericks et al. [65]. The mobile phase consisted of water/formic acid/acetonitrile (87:10:3, *v*/*v*/*v*; eluent A) and water/formic acid/acetonitrile (40:10:50, *v*/*v*/*v*; eluent B). The gradient programme was as follows: from 10% to 25% B (10 min), from 25% to 31% B (5 min), from 31% to 40% B (5 min), from 40% to 50% B (10 min), from 50% to 100% B (10 min), 100% B isocratic (5 min), and from 100% to 10% B (1 min). 

Anthocyanins were quantified using calibration curves generated from Cyanidin-3-glucoside (Cya 3-glc) and Pelargonidin-3-glucoside (Pel 3-glc) standards. Pelargonidin-3-rutinoside (Pel 3-rut) was quantified using the Pel 3-glc calibration. All anthocyanins were calculated as milligrams per gram of dry weight (mg/g DW).

### 4.4. Statistical Analysis

Data were subjected to analysis of variance (randomized complete design; ANOVA). Differences between mean values were assessed by Tukey honest significant difference (HSD) with the analytical software STATISTIX 9.0 (Analytical Software, Tallahassee, FL, USA). Prior to ANOVA, normality and homogeneity were tested by the Kolmogorov–Smirnoff test and Cochran’s *C* test, respectively. Pearson’s correlation and regression analysis were performed to analyse the relationship between variables.

## 5. Conclusions

It has long been known that strawberries provide benefits for human health, but the present study highlights that, despite strawberry achenes representing a small fraction of the fruit, their contribution to total fruit antioxidant content and capacity is very important, providing a new profitable product for improving human health. Moreover, this study revealed that strawberry antioxidant compounds are compartmented in the fruit, achenes having different quantity and quality, and enriched in non-flavonoids, pointing to differences in the ability for radical scavenging between flesh and achenes. Our results showed that commonly used extraction methods underestimate antioxidant quantity, and that in vitro digestion is a good approximation to test how antioxidants are released from different food matrices and are able to be incorporated into the bloodstream under physiological conditions. This study reveals the importance of considering achenes in breeding programs and in further studies on strawberry varieties at different environmental ranges, since strawberry fruit antioxidant composition varies depending on the cultivars and environmental conditions [55].

## Figures and Tables

**Table 1:** Estimation of relative contribution (%) of total phenolic compounds (TPCs), flavonoids (TFCs) and anthocyanins (ACs) and antioxidant capacity by FRAP, DPPH and TEAC in non-digested flesh and achenes on a whole fruit.

	TPC	TFC	AC	FRAP	DPPH	TEAC
Flesh	59	77	92	47	55	19
Achene	41	23	8	53	45	81

**Table 2:** Content of total phenolics (TPCs; mg GAE/g DW), total flavonoids (TFCs; mg CE/g DW), and total anthocyanins (ACs; mg Pel-glc/g DW), and antioxidant capacity (in mg TE/g DW), determined by FRAP, DPPH and TEAC in flesh (F) and achenes (A) extracts before (non-digested) and after gastric (gastric Fraction) and total digestion (intestinal fraction). All analyses were performed in triplicate. Data represent the mean ± Standard error (SE). Post hoc comparisons were done by Tukey’s test. (*) and (^ns^) indicate significant (*p* < 0.05) and non-significant differences, respectively.

	Non-Digested		Gastric Fraction		Intestinal Fraction		Ee ^1^		R (%) ^2^	
	Flesh	Achene	Flesh	Achene	Flesh	Achene	Flesh	Achene	Flesh	Achene
TPC	37.68 ± 0.95	382.87 ± 4.69 *	255.02 ± 4.40	600.34 ± 33.51 *	41.17 ± 0.61	16.55 ± 0.39 *	6.8	1.6	16.1	2.8
TFC	22.04 ± 0.49	93.92 ± 4.70 *	135.32 ± 4.08	133.66 ± 9.13 ^ns^	14.40 ± 0.13	7.68 ± 1.60 ^ns^	6.1	1.4	10.6	5.7
AC	5.4 ± 0.78	6.97 ± 0.79 ^ns^	41.58 ± 5.37	17.59 ± 2.42*	0.00 ± 0.00	0.00 ± 0.00 ^ns^	7.7	2.5	0.0	0.0
FRAP	149.40 ± 18.69	2474.22 ± 38.92 *	1254.45 ± 46.66	4522.91 ± 223.18 *	101.31 ± 1.87	75.25 ± 1.95 *	8.4	1.8	8.1	1.7
DPPH	100.54 ± 4.49	1192.21 ± 62.09 *	119.25 ± 14.30	3931.60 ± 771.70 *	90.04 ± 4.58	32.85 ± 2.15 *	1.2	3.3	75.5	0.8
TEAC	20.13 ± 7.64	1286.39 ± 110.39 *	2174.79 ± 46.74	2100.68 ± 298.81 ^ns^	286.82 ± 2.79	86.57 ± 4.69 *	108.0	1.6	13.2	4.1

^1^ Ee: Extraction efficiency of gastric conditions (gastric fraction vs. non-digested extracts); ^2^ R (%): % recovery of compounds from gastric to intestinal fraction.

**Table 3:** Content (mg/g DW) of phenolic acids (caffeic acid, chlorogenic acid and ellagic acid) and anthocyanins (Cyanidin 3-glucoside, Pelargonidin 3-glucoside and Pelargonidin 3-rutinoside) in flesh (F) and achene (A) extracts before (non-digested) and after gastric (gastric fraction) and total digestion (intestinal fraction). All analyses were performed in triplicate. Data represent the mean ± Standard Error (SE). Post hoc comparisons were done by Tukey’s test. (*) and (^ns^) indicate significant (*p* < 0.05) and non-significant differences, respectively.

	Non-Digested		Gastric Fraction		Intestinal Fraction		Ee ^1^		R (%) ^2^	
	Flesh	Achene	Flesh	Achene	Flesh	Achene	Flesh	Achene	Flesh	Achene
**Phenolic acids**										
Caffeic acid	0.45 ± 0.04	0.40 ± 0.03 *	0.28 ± 0.02	0.21 ± 0.02 ^ns^	0.16 ± 0.01	0.49 ± 0.04 *	0.6	0.5	58.3	236.6
Chlorogenic acid	1.42 ± 0.12	1.22 ± 0.11 *	1.25 ± 0.11	1.06 ± 0.09 ^ns^	0.57 ± 0.05	0.75 ± 0.07 ^ns^	0.9	0.9	45.5	70.6
Ellagic acid	1.26 ± 0.11	2.09 ± 0.18 *	0.77 ± 0.07	0.71 ± 0.06 ^ns^	0.19 ± 0.02	0.35 ± 0.03 *	0.6	0.3	24.4	48.6
**Anthocyanins**										
Cyanidin 3-glc	0.65 ± 0.07	1.98 ± 0.11 *	1.98 ± 0.11	2.57 ± 0.01 *	0.00 ± 0.00	0.76 ± 0.11 *	3.0	1.3	0.0	29.5
Pelargonidin 3-glc	4.10 ± 0.36	8.55 ± 0.07 *	6.28 ± 0.11	13.55 ± 0.88 *	0.90 ± 0.07	1.43 ± 0.22 ^ns^	1.5	1.6	14.3	10.5
Pelargonidin 3-rut	0.51 ± 0.04	0.32 ± 0.05 ^ns^	1.85 ± 0.33	4.26 ± 0.18 *	0.44 ± 0.02	0.00 ± 0.00 *	3.6	13.2	24.0	0.0

^1^ Ee: Extraction efficiency of gastric conditions (Gastric fraction vs. non-digested extracts); ^2^ R (%): % recovery of compounds from gastric to intestinal fraction.

**Table 4:** Regression coefficients and *p*-values of TPCs, TFCs, and total ACs and antioxidant capacity by three different assays (DPPH, FRAP and TEAC).

	DPPH	FRAP	TEAC
TPC	0.507	0.944	0.657
	0.010	0.005	0.010
TFC	0.146	0.641	0.900
	0.020	0.005	0.010
AC	0.002	0.080	0.545
	0.246	0.060	0.010
